# Rapid antibiotic susceptibility testing and species identification for mixed samples

**DOI:** 10.1038/s41467-022-33659-1

**Published:** 2022-10-20

**Authors:** Vinodh Kandavalli, Praneeth Karempudi, Jimmy Larsson, Johan Elf

**Affiliations:** Dept. Cell and Molecular Biology, Uppsala University, Uppsala, Sweden

**Keywords:** Microbiology techniques, Image processing, Antimicrobial resistance

## Abstract

Antimicrobial resistance is an increasing problem on a global scale. Rapid antibiotic susceptibility testing (AST) is urgently needed in the clinic to enable personalized prescriptions in high-resistance environments and to limit the use of broad-spectrum drugs. Current rapid phenotypic AST methods do not include species identification (ID), leaving time-consuming plating or culturing as the only available option when ID is needed to make the sensitivity call. Here we describe a method to perform phenotypic AST at the single-cell level in a microfluidic chip that allows subsequent genotyping by in situ FISH. By stratifying the phenotypic AST response on the species of individual cells, it is possible to determine the susceptibility profile for each species in a mixed sample in 2 h. In this proof-of-principle study, we demonstrate the operation with four antibiotics and mixed samples with combinations of seven species.

## Abstract (web summary)

Rapid antibiotic susceptibility testing (AST) is needed. Here the authors report a method for phenotypic AST at the single cell level, using a microfluidic chip that allows for subsequent genotyping with in situ FISH; they apply this to a mixed sample of 7 species and 4 antibiotics.

## Introduction

The rapid increase in antibiotic resistance is a serious threat to human health; access to effective antibiotics is a cornerstone of modern medicine and a prerequisite for e.g. cancer treatment and surgery. Different investigations^1,2^ make different estimations of how grave the situation is but there is a consensus view that action needs to be taken or the costs both in terms of human suffering and global economic impact will be staggering^3^. Experts also agree that the problem is at least partly due to indiscriminate use and misuse of a wide range of antibiotics^4^. To overcome this problem, personalized and rapid antibiotic susceptibility tests (ASTs) are needed, ideally at the point of care^5^. Without these tools, physicians are left with no other option than to prescribe broad-spectrum antibiotics in many cases since it can take several days to identify the pathogen(s) and the resistance profile.

The limitations of conventional phenotypic ASTs (disk diffusion agar dilution or broth microdilution) are that they require bacterial growth for extended periods in the presence and absence of antibiotics to see an effect. However, for certain types of bacterial infections, even a delay of 6 h before treatment is initiated can have severe consequences^6^. One such example is sepsis, where the risk of death has been estimated to increase by 7.6% for every hour that effective treatment is not given^7^. Further, it is shown that in the absence of fast AST, more than 25% of septic patients were treated by clinicians with inappropriate antibiotics, which is strongly associated with mortality^8–10^. Thus, rapid and accurate ASTs are needed to save lives. But considering the increases in AMR, life-threatening conditions are not the main culprit, but rather the bulk usage of antibiotics for more benign conditions^11^. The exhaustion of effective antibiotics is also driven by the strategy to change first-line antibiotics when the local resistance prevalence has reached approximately 10–20%. If fast, reliable ASTs were accessible, high-resistance antibiotics could be used for 80–90% of the infections that are still susceptible.

The obvious need and benefits of rapid AST, both for saving lives and guiding prescriptions, have resulted in the development of several new methods over the last decade. These methods are described in several recent reviews, e.g.,^4,12^, and we will not repeat all the pros and cons of the different methods here. Briefly, these methods can be divided into two categories, genotypic and phenotypic. Genotypic methods identify specific genetic markers that are associated with antibiotic resistance. Although these methods can rapidly detect the presence of specific resistance genes, they depend on our knowledge of the resistance mechanisms which is far from complete^13^, in particular considering the rapid emergence of new resistance mechanisms. Furthermore, the absence of resistance genes does not predict susceptibility to antibiotics^14^, i.e., you may learn what not to use but not what will work. In phenotypic methods, the bacteria are exposed to an antibiotic and their phenotypic response, e.g., lysis or growth rate reduction, is monitored. Phenotypic methods work irrespective of the mechanism of resistance. If the phenotypic response is there, the bacterium is susceptible. The various rapid phenotypic ASTs that have reached the market can deliver an answer (susceptible or resistant) in 2–6 h for positive blood cultures that have been growing >6 h from sampling the patient. For other samples, e.g., urine, the time-to-answer can be reduced to 30 min for gram-negative species by loading the sample directly into a microchip and measuring the growth rate with and without antibiotics^15^.

A limitation of all rapid phenotypic AST methods is that they only work if the species of the bacteria are known. Detailed species ID is not needed for infections with a narrow spectrum of pathogens^16^, but for sepsis and other more complex infections, it is essential. MALDI-TOF mass spec is currently the golden standard for species determination^17^. MALDI-TOF does however still require pre-culture of single bacterial species and may not work well for mixed infections commonly seen in sepsis, wounds, catheter-associated UTIs^18^, and community-acquired pneumonia, among others^19^. The challenge remains to make rapid phenotypic AST with species ID.

**Fig. 1:**
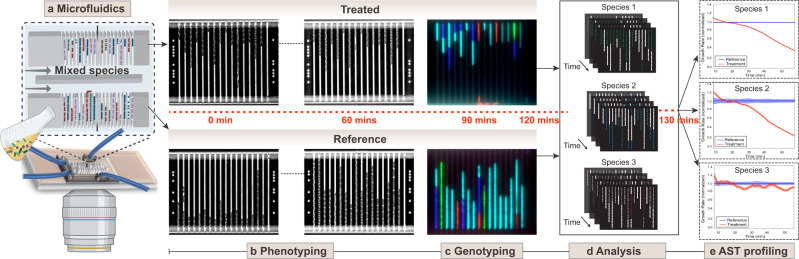
Schematic representation of the AST workflow with timeline. **a** A cartoon of the microfluidics setup with the mixed species loaded on the chip. **b** Time-lapse phase-contrast images of the cells in the traps when grown in media with (top) and without (bottom) antibiotics. **c** Fluorescence images of the bacteria with ssDNA probes targeting the ribosomal RNA of specific bacteria for species identification. **d** Analysis of time-lapse stacks and species ID using deep learning for segmenting and tracking cells. **e** Detection of AST profiles for individual pathogens at a given antibiotic concentration. Part of Fig. 1a created using www.biorender.com.

To address this issue, we use a microfluidic chip to rapidly capture individual bacteria from a sample and optically monitor their growth with and without antibiotics. Next, we identify the bacterial species by fluorescence in situ hybridization (FISH) with species-specific ssDNA probes targeting the 16s/23s rRNA sequence. Once we have the species ID and AST response for each bacterium in the microfluidic chip, we stratify the AST response based on species, which implies that we can have samples with mixed species. A schematic overview of the approach is presented in Fig. 1.

In this proof of principle application, we perform the ASTs for seven common pathogens (*Escherichia coli*, *Klebsiella pneumonia, Pseudomonas aeruginosa, Proteus mirabilis*, and *Acinetobacter baumannii*, as examples of gram-negative strains, and *Enterococcus faecalis* and *Staphylococcus aureus* as examples of gram-positives). We test four different antibiotics from different classes: Vancomycin (Van) [Glycopeptide], Ciprofloxacin (CIP) [Fluoroquinolones], Gentamicin (Gen) [an aminoglycoside], and Nitrofurantoin (NIT) [other agents]. Finally, we show how single-round, multi-color labeling enables the identification of up to ten species simultaneously.

## Results

### Phenotypic AST followed by Genotyping by FISH

The culture chip that we have developed for this assay is capable of rapid capture of bacteria directly from a liquid sample and allows for optical monitoring of bacterial growth with and without antibiotics in real time. The same chip design has previously been used to capture bacteria from blood cultures despite an overwhelming excess of blood cells^20^. The chip features two rows of 3000 cell traps each. Each trap measures 1.25 × 1.25 × 50 μm^15^ and has a constriction at the end which prevents the bacteria from escaping the trap while still allowing media and probes to flow around the cells. To simulate a mixed infection situation, bacterial overnight cultures of several different species were diluted in a Mueller Hinton (MH) broth, pooled, and directly loaded into the microfluidic chip. In a typical experiment, loading one or a few cells in each trap takes 1 min at ~10^5^ CFU/ml. We supplied growth media with antibiotics to the traps in one of the two rows and plain growth media in the other.

The phenotypic response to the antibiotic was determined in ≈60 min by capturing 100X phase-contrast images of each cell every 2 min and calculating the growth rates of individual cells in 10 min sliding windows. The phenotypic response can be pushed to shorter times depending on which antibiotics are used and at which concentration^15^. To identify the species of each bacterial cell, we performed fluorescence in situ hybridization (FISH) using species-specific, fluorescent ssDNA probes. These probes (Supplementary Data 1) bind to the abundant 16s/23s ribosomal RNA sequences and have previously been successfully used for species identification in positive blood cultures^21^. The species classification method for FISH signals is described in SI method 3.

### Analysis using Deep learning models

**Fig. 2:**
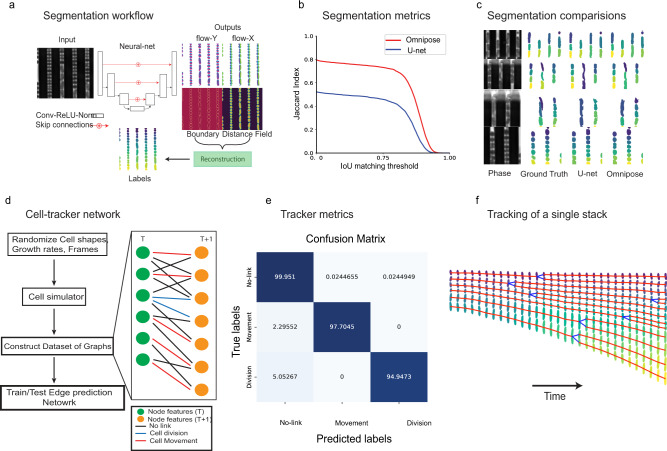
Analysis. **a** Omnipose network showing network structure, inputs, and outputs to the neural network and reconstruction of output to generate cell masks. **b** Quantified performance of cell segmentation in mother-machine devices. Average precision vs IOU threshold plot for mixed-species dataset. The IOU threshold defines valid matching between predicted mask and ground-truth masks, 0.5 indicating half the pixels were correctly matched and 1 indicating pixel perfect match for every cell. Average precision (TP / (TP + FP + FN)) is calculated from the valid matches (TP), no valid matches (FP), and the ground-truth masks that have no valid match (FN). **c** Few examples of phase-contrast images, ground-truths and network predictions of U-net and Omnipose. **d** Overview of tracker network. **e** Confusion matrix of the edge predictions. **f** Tracks of cells in one channel of the mother-machine device.

Performing growth-rates analysis on multi-species samples requires a general method for detecting and tracking cells that come in different shapes and sizes. In Delta 2.0^22^, a U-net^23^ was used to predict cell-vs-background maps of phase-contrast images and showed excellent performance for *E. coli* cells, both in mother-machine-like devices and on agarose pads. More general algorithms for cell segmentation, such as Cellpose^24^ and its successor Omnipose^25^ construct cell identities from intermediate fields (Fig. 2a) learned by a convolutional neural network, similar to a U-net. Omnipose was shown to perform segmentation of bacterial cells independently of their morphology. Approaches that calculate cell identities (Cellpose, Omnipose) assign different unique labels to cells that share a boundary while cell-vs-background approaches (U-net) can not be used to separate cells that share a boundary. The former approaches require cell labels as training data while the latter needs only binary masks.

A ground-truth training dataset (82 images) of mixed cells growing in mother-machine devices was created with human supervision using the LabelsToROIs tool^26^ and custom scripts (SI method 1). We use an Omnipose model trained on this data for cell segmentation. Training and performance of Omnipose and U-net are also described in SI method 3. The run-time performance of reconstruction steps from Omnipose network outputs was improved two-fold (SI Fig. 1b). Omnipose showed superior performance (Fig. 2b) on mixed-species data compared to a U-net trained to predict binary masks. We showed that the omnipose performed equally well on mixed-species data as on data from pure *E. coli* cultures (SI Fig. 1b, e); the performance of U-net, on the other hand, depended on the species. Figure 2c shows phase contrast images, their corresponding ground truths, and cells detected using Omnipose and U-net approaches, respectively.

Previous approaches to successful *E. coli* tracking have constructed cell lineages using scoring mechanisms based on overlapping regions^27^ or mother-daughter binary-mask predictions using the full features from the images^22^. The former approach is very sensitive to tuning overlap parameters while the latter is computationally expensive when a lot of small cells are present in the data. Inspired by recent developments in using Siamese networks^28^ and graph formulations^29^ for general object-tracking, we have now developed an approach that performs cell tracking using a network that compares cell properties between two frames and predicts the presence and type of connections, e.g., cell growth or division event (Fig. 2d). Training data for this network was obtained using a simulator (SI method 4) that simulates cells of various kinds and growth rates growing in mother-machine channels. The training of the tracking network is described in the Supplementary information (SI method 4). In Fig. 2e, we show the confusion matrix of the model used for tracking experimental data evaluated on 100 randomly generated time-series stacks. Before constructing the tracks, the links that have abrupt increases in areas were removed as they correspond to merge events caused by segmentation errors (SI method 4). Figure 2f (left) shows a single segmented mother-machine trap tracked through time with growth/movement links (red) and division links (blue). Species assignment to tracks based on the species ID identified in the last frame was performed after cell-tracking (SI method 5).

### Species-wise AST response

**Fig. 3:**
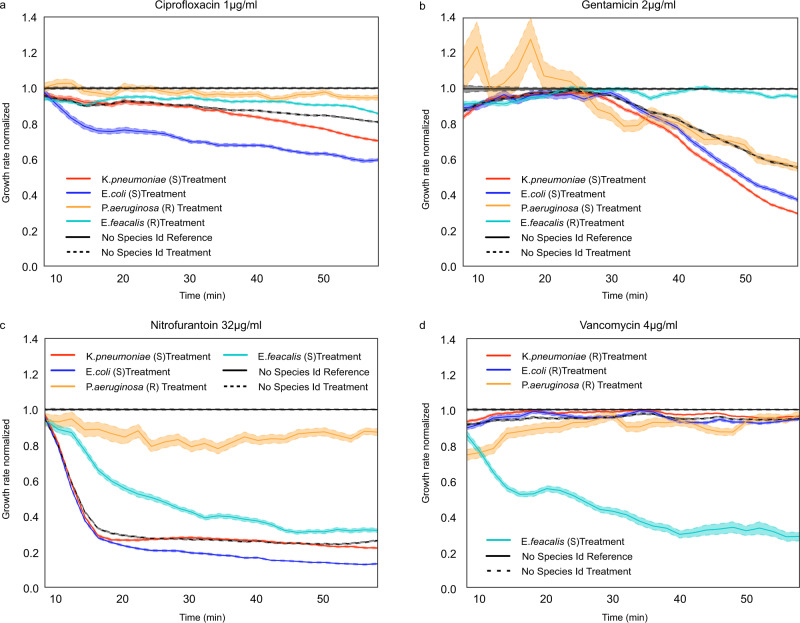
Species stratified responses to antibiotic treatments. **a**–**d** AST profiles with normalized growth rates for the four antibiotics used. The species stratified responses (mean and SEM), as well as the pooled response (without species stratification), are shown for each antibiotic. In all AST profile plots, S and R represent the Susceptible and Resistance, respectively. The experiment is performed once per antibiotic, although several non-reported experiments were performed for calibration.

With the species ID and AST response for each cell in the microfluidic chip, the species-specific AST response could be determined in the mixed samples. Here, we first demonstrate the capability of the method to characterize a mixed sample of four different species, although clinical patient samples are more likely to contain only one or two^30–32^. The AST responses are shown in Fig. 3a–d. In each experiment, we obtained growth-response curves for three gram-negative strains (*Escherichia coli*, *Klebsiella pneumoniae*, and *Pseudomonas aeruginosa)* and one gram-positive strain (*Enterococcus faecalis)*. The experiments were performed with four different antibiotics: Vancomycin (Van) [Glycopeptide], Ciprofloxacin (CIP) [Fluoroquinolones], Gentamicin (Gen) [an aminoglycoside], and Nitrofurantoin (NIT) [other agents]. We present the results as response plots from individual experiments to simulate the clinical-sample situation. For comparison, we also display the average responses that would have been the result of the growth-rate measurements without species information. Successful AST profiling was achieved with samples containing as few as 100 bacteria/species. We used bacteria without specific resistance genes, but since some species have a natural resistance to specific antibiotics, the AST response varied with the species. For example, *Pseudomonas* species are naturally resistant to Ciprofloxacin (Fig. 3a) and Nitrofurantoin (Fig. 3b), and their growth remained unaffected in the presence of 1 μg/ml Ciprofloxacin or 32 μg/ml Nitrofurantoin. The growth rate of the other bacterial species dropped more than 10% in 30 min in the presence of these drugs. Importantly, we see from the average, non-species-stratified data that without access to species ID, we would not have been able to detect the resistant *Pseudomonas* in the mixed population. Similarly, as expected, all species but *E. faecalis* were found to be susceptible to Gentamicin (2 μg/ml) (Fig. 3c) and resistant to Vancomycin (4 μg/ml) (Fig. 3d).

### Scaling up FISH probing to 10 species

**Fig. 4:**
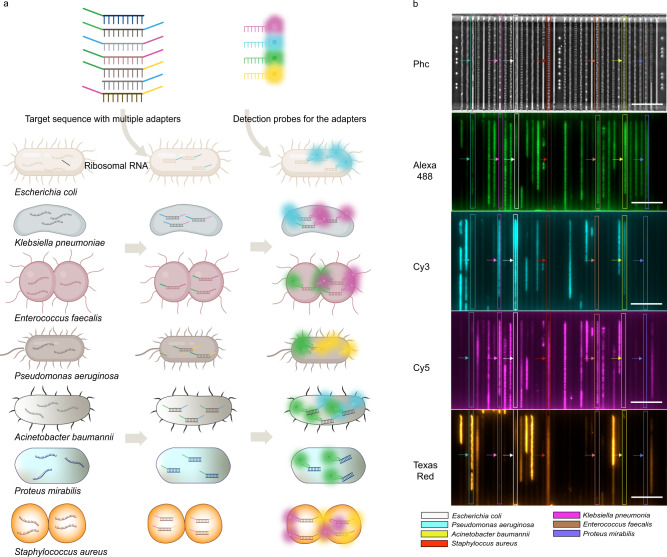
Combinatorial FISH. **a** Overview of the combinatorial FISH probing for the multi species identification. A cartoon illustrating the different bacterial species with their ribosomal RNA (left). Illustration of the specific sequences with the multiple adapters targeting the ribosomal RNA of individual bacteria and its hybridization to the target rRNA (middle). Detection probes with different fluorophores. Hybridization of detection probes to the adapter sequences along with unique sequences that are targeted to the species specific rRNA (Right). **b** Example images (Scale bar 20 µm) of mixed species loaded in the microfluidic chip and probed using combinatorial FISH for species identification. After the hybridization step, cells were imaged in different channels (PhC, Alexa 488, Cy3, Cy5, and Texas Red). The bacterial species are marked in white (*Escherichia coli*), magenta (*Klebsiella pneumoniae*), cyan (*Pseudomonas aeruginosa*), brown (*Enterococcus faecalis*), yellow (*Acinetobacter baumannii*), navy blue (*Proteus mirabilis*) and red (*Staphylococcus aureus*). The experiment with all seven species mixed was performed a single time. However, similar experiments with all adaptors and probes mixed are reported in Fig. 5.

**Fig. 5:**
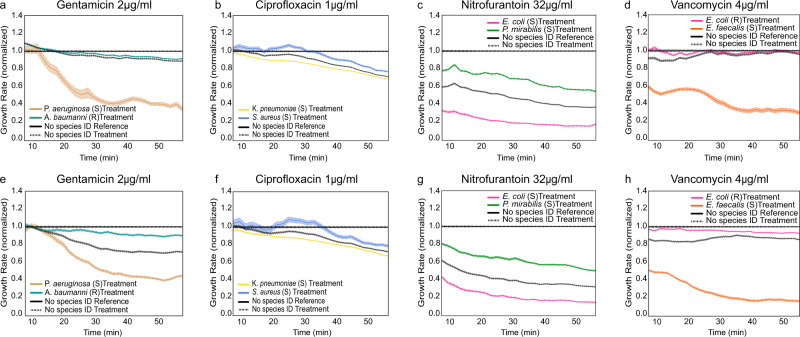
Species-wise AST profiles for experiments with 2 species using the combinatorial FISH method. **a** *P. aeruginosa* and *A. baumannii* were treated with Gentamicin, **b** *K. pneumoniae*, and* S. aureus* treated with Ciprofloxacin, **c** *E. coli*, and *P. mirabilis* treated with Nitrofurantoin, **d** *E. coli* and *E. faecalis* treated with Vancomycin. **e**–**h** Biological repeats of 5a-5d, respectively. In all AST profile plots, S and R represent Susceptible and Resistance, respectively. Normalized growth rates ± SEM as a function of time are shown for each species detected in the experiments.

It is estimated that >90% of clinical sepsis samples feature a subset of the 10 most frequent bacterial pathogens^33^. To increase the number of species that we can identify, we performed combinatorial FISH using a species-specific adaptor that can bind two different fluorescent oligo probes. With probes of four different colors, this set-up can identify up to 10 species (Fig. 4a). We demonstrated the combinatorial FISH approach by identifying seven species loaded in the culture chip (Fig. 4b). Species-wise combination of signals using all the adapters and probes are shown in SI Fig. 7. Species classification was done using a random-forest classifier on the 4-d signal obtained from stacking images of four fluorescence channels (SI method 6). To demonstrate species-wise AST profiles using combinatorial FISH, we performed experiments with combinations of two species treated with different antibiotics (Fig. 5). For example, when *Escherichia coli* and *Enterococcus faecalis* were treated with Vancomycin (4 μg/ml), the two species showed clear, distinguishable growth-rate profiles corresponding to resistance and susceptibility, respectively (Fig. 5d). Repeats for these experiments are shown in Fig. 5e–h.

## Discussion

In conclusion, we have demonstrated that it is possible to make rapid AST for mixed-species samples by performing sequential single-cell phenotypic susceptibility testing and fluorescence in situ hybridization in a microfluidic chip. Importantly, ID determination is also useful for non-mixed samples when it is essential to know which MIC breakpoints to use for making the SIR (susceptible-intermediate-resistant) call. Although species determination for non-mixed samples is possible by MALDI-TOF, it requires many more bacterial cells than direct single-cell imaging.

Since antibiotic susceptibility breakpoints of closely related species are often the same, it can sometimes be enough to distinguish the bacterial family or genera. For example, an Enterobacteriales-specific color ID-code to cover *Escherichia, Klebsiella*, and *Salmonella*, which have very similar resistance breakpoints^34^. Different species-specific probes would in this case map the same color ID code, such that the ten codes can be used more efficiently. Similarly, we expect that it would be possible to have a common ID code for contaminant species, such that they are not mistaken for pathogens. However, if more than 10 specific ID codes are required, stripping and reprobing allow an exponential increase in how many classes can be identified^35,36^, but at the expense of time.

In the current implementation, we ran the test one concentration at a time on a high-end research microscope and post-processed the imaging data. To make the technology useful in a clinical setting, data should be analyzed during the experiment and the fluidic chip should be parallelized to run multiple antibiotic concentrations simultaneously. Although we have not tested this type of setup in the present study, it should allow a MIC determination to identify intermediate isolates. A system with five different antibiotics at five different concentrations and a high level of automation has been developed for uncomplicated urinary tract infections^20^, which shows that upscaling to multiple testing conditions is possible. Finally, we would like to emphasize that the present study is proof of principle of combined phenotypic AST and species identification at the single-cell level. For this method to be clinically relevant, it needs to be calibrated and tested on many more clinical isolates as well as on real patient samples.

## Methods

### Bacterial strains and antibiotics

In this study, as a gram-negative representative we used *E*. *coli* K12 MG1655 (DA4201), *K*. *pneumoniae* (ATCC 13883), A. *baumanni* (DA68153), P. *mirabilis* (ATCC 29906) and *P*. *aeruginosa* (DA6215). As a gram-positive representative, we used *E*. *faecalis* (ATCC 51299) *and S. aureus* (ATCC 29213). We also used the fluorescently tagged *P. aeruginosa* (PAO1-GFP) cells, a kind gift from Oana Ciofu^37^. Antibiotics (Ciprofloxacin, Gentamicin, Nitrofurantoin, and Vancomycin) were purchased from Sigma-Aldrich. Stock solutions were prepared as per the supplier guidelines and stored at −20 °C. The solutions were thawed to room temperature before performing the AST experiments. We used concentrations of the antibiotics corresponding to the MIC values for *E. coli* (ATCC 25922) as defined by the European Committee on Antimicrobial Susceptibility Testing (EUCAST). We note that another, higher, concentration may be optimal to make a rapid SIR call.

### Media and culture conditions

In all experiments, Mueller-Hinton (MH) medium (70192; Sigma-Aldrich) was used as a broth. Overnight cultures (ONC) were prepared by inoculating bacteria from glycerol stocks (−80 °C) in MH medium and incubating at 37 °C for 14–15 h with continuous shaking (200 rpm). From ONC, cells were diluted 1:1000 times into fresh MH medium supplemented with a surfactant (Pluronic F-108; 542342; Sigma-Aldrich; 0.085% (wt/vol) final concentration). The liquid culture was grown by shaking at 200 rpm at 37 °C for 2 h. Next, to perform AST experiments, we mixed the different strains in equal concentrations and loaded them on the microfluidic chip.

### The microfluidic chip and setup

The chip consist of mainly two parts: a micromolded silicone elastomer [Sylgard 184; polydimethylsiloxane (PDMS)] and a 1.5 glass coverslip (Menzel-Gläser) which are covalently bonded together. Chip design and preparation were previously described in^15,38^. These references also describe the numbering of the ports used below. After punching the ports on the chip, it was placed on the microscope, and tubing (TYGON) was connected with a metal tubing connector. Briefly, cells were loaded using port 8.0 and port 2.0 was used for the exchange of medium with the probes. Ports 5.1, 5.2, and 6.0 were used for maintenance of back-channel pressure, and ports 2.1 and 2.2 were used for the supply of MH medium with and without the antibiotics at a pressure of 200 mbar. The pressure was controlled by an OB1-Mk3 regulator (Elveflow).

### Microfluidic experiments

Imaging starts within five minutes after the supply of medium with and without the antibiotics to the cells. We used a Nikon Ti2-E inverted microscope equipped with a Plan Apo Lambda 100× oil immersion objective (Nikon). Images were captured by the Imaging Source (DMK 38UX304) camera. For phase contrast and fluorescence images, we used the optical setup as previously described in^15,38^ and controlled by Micro-Manager^39^, and an in-house built plugin. We maintained 30 °C using a temperature controllable unit and a lexan enclosure (Oklab).

### Fast phenotypic AST

The cells were loaded on the chip and exposed to growth media with and without the antibiotics in two different rows. In each row, a total of 70–80 positions, each including 16–21 cell traps, were images in the phase contrast channel (30 ms exposure time) every two minutes for an hour.

### Genotyping

After phenotyping, the medium from ports 2.1 and 2.2 were depressurized to zero. To fix the cells, formaldehyde (4%) was added by switching the medium and applying pressure 200 mbar from port 2.0 for 4 min and subsequently washing the cells with 1 × phosphate-buffered saline (PBS) for 3 min. Cells were permeabilized by 70% EtOH for 4 min and washed with 1 × PBS (3 min). Cells were next treated with lysozyme (2 mg/ml) and lysostaphin (0.1 mg/ml) for 3 min and followed by quick washing with 1 × PBS for another 3 min. (Lysostaphin is only needed for *S. aureus* and was not used in Fig. 3). For species identification, we pooled all specific ssDNA probes (0.1 μM) (Supplementary Data 1) in a hybridization solution (30% formamide and 2 × SSC) and hybridized them for 30 min at 30 °C. In the case of the combinatorial method, we pooled all the detection probes (0.1 μM) (Supplementary Data 2) and species-specific adaptor sequences (Supplementary Data 3) in a hybridization solution (30% formamide and 2 × SSC) and hybridized them for 60 min at 30 °C. Next, we captured the fluorescence images for each probe in different channels (TYE 665, TYE 563, Texas Red, and Alexa Fluor 488) at 300 ms exposure times and respective phase contrast images at 30 ms exposure time. In total, it took 10–15 min to image all the positions in all the channels on the chip.

### Cell segmentation and channel detection

Phase-Contrast images of the cells growing in channels were segmented for both cells and channels using a deep learning model with Omnipose method. The cell-segmentation model was trained with data obtained from manually-labeled images of mixed species data and data obtained from *E. coli* (K12 MG1655 intC::P70-venusFast) that constitutively expresses mVenus. The training procedure was enhanced with data augmentations to force learning outputs of Omnipose network in different orientations and scales. The model training and performance compared to U-net is described in SI method 1. The segmentation network performance on different species is also shown in SI method 1. The channel segmentation model was trained with data that was refined based on histogram profiles, also described in SI method 2. After obtaining channel locations, time-series stacks of segmented cells and corresponding fluorescent channel images were bundled for tracking, species assignment, and growth rate calculations.

### Cell tracking

Cells were tracked through time using a neural network that scores links between cells from one frame to the next and predicts the type of link between them. Training data for cell tracking was obtained by simulating the growth of cells in mother-machine channels and is described in SI method 4. Cells of different shapes, sizes, and growth rates were used to generate ground truths for the tracking network. Tracker network training and its performance are described in SI method 4. The predictions of the network are cleaned for error using IoU (Intersection-over-Union) metric to remove spurious predictions. At test time, cells between frames were linked based on the probability scores, and tracks were generated by chaining a series of links. Cell tracks were corrected for errors (SI method 4).

### Species assignment and growth curve splitting

For experiments where a single probe binds to a single species, fluorescent images for each mother machine channel were corrected for background, and continuous regions of signal above thresholds were mapped to species labels (SI method 3). Experiments with multiple fluorescent probes for a single species were classified using a Random-Forest classifier, described in SI method 6. Continuous regions corresponding to one species (>90 pixels) were identified and bounding boxes were drawn around these regions. Cell tracks falling in these regions in the last frame before fixing cells were labeled with the corresponding species and all the species labels were rolled back to time point 0. Growth rates were calculated by fitting exponential curves on the areas of cells in a moving 5 timepoint window (SI method 7).

### Oligos and probes design

FISH probe sequences for the individual target rRNA were obtained from the probeBase^21,40,41^ and purchased from Integrated DNA Technologies (www.idt.com), see Supplementary data 1. For the combinatorial FISH probing, we used barcode sequence and detection probes, which are listed in Supplementary Data 2–4, purchased from IDT.

### Statistics and reproducibility

All experiments were conducted using the same set-up. Both treated and reference cells used the same segmentation and tracking models. The experiments were not randomized. For growth rate measurements, each species’ growth rate was calculated using at least 100 cells. Cells with segmentation errors were not included in the growth-rate calculations. All AST profiles show normalized growth rates ± SEM. Detailed information on experimental repeats can be found in the individual figure legend.

### Reporting summary

Further information on research design is available in the Nature Research Reporting Summary linked to this article.

## Supplementary information

Supplementary Information

Reporting Summary

Description of Additional Supplementary Files

Supplementary data 1

Supplementary data 2

Supplementary data 3

Supplementary data 4

Supplementary data 5

Supplementary movie S1

Supplementary movie S2

Supplementary movie S3

Supplementary movie S4

Supplementary movie S5

## Data Availability

Raw microscopy data for all experiments shown in the paper are available at 10.17044/scilifelab.20969161. All analysis objects and code are also available at 10.17044/scilifelab.20969161. All strains will be provided upon request to J.E. Source data are provided with this paper.

## Source data

Source data

## Code availability

The code used for analysis and reproducing all the figures is available at https://github.com/karempudi/ASTFISH.git. Weights of trained neural networks for cell segmentation and tracking are available at 10.17044/scilifelab.20969161.

## References

[1] GBD 2016 Causes of Death Collaborators (2017). Global, regional, and national age-sex specific mortality for 264 causes of death, 1980–2016: A systematic analysis for the Global Burden of Disease Study 2016. Lancet.

[2] O’neill. Antimicrobial resistance: Tackling a crisis for the health and wealth of nations. Review on antimicrobial resistance. https://amr-review.org/ (2014).

[3] O’Neil, J. Tackling Drug-resista*nt Infections Globally: Final Report and Recommendations*. https://books.google.com/books/about/Tackling_Drug_resistant_Infections_Globa.htmlhl=&id=aa6lAQAACAAJ (2016).

[4] van Belkum A (2020). Innovative and rapid antimicrobial susceptibility testing systems. Nat. Rev. Microbiol..

[5] Schoepp, N. G. et al. Rapid pathogen-specific phenotypic antibiotic susceptibility testing using digital LAMP quantification in clinical samples. *Sci. Transl. Med*. **9**, eaal3693 (2017). 10.1126/scitranslmed.aal3693 PMC6765391 28978750

[6] Bernhard M, Lichtenstern C, Eckmann C, Weigand MA (2014). The early antibiotic therapy in septic patients–milestone or sticking point?. Crit. Care.

[7] Kumar A (2006). Duration of hypotension before initiation of effective antimicrobial therapy is the critical determinant of survival in human septic shock. Crit. Care Med..

[8] Ibrahim EH, Sherman G, Ward S, Fraser VJ, Kollef MH (2000). The influence of inadequate antimicrobial treatment of bloodstream infections on patient outcomes in the ICU setting. Chest.

[9] Caliendo AM (2013). Better tests, better care: Improved diagnostics for infectious diseases. Clin. Infect. Dis..

[10] Kumar A (2009). Initiation of inappropriate antimicrobial therapy results in a fivefold reduction of survival in human septic shock. Chest.

[11] Ventola CL (2015). The antibiotic resistance crisis: part 1: Causes and threats. P T.

[12] Vasala A, Hytönen VP, Laitinen OH (2020). Modern tools for rapid diagnostics of antimicrobial resistance. Front. Cell. Infect. Microbiol..

[13] Cockerill FR (1999). Genetic methods for assessing antimicrobial resistance. Antimicrobial Agents Chemother..

[14] Bard JD, Lee F (2018). Why can’t we just use PCR? The role of genotypic versus phenotypic testing for antimicrobial resistance testing. Clin. Microbiol. Newsl..

[15] Baltekin, Ö., Boucharin, A., Tano, E., Andersson, D. I. & Elf, J. Antibiotic susceptibility testing in less than 30 min using direct single-cell imaging. *Proc. Natl Acad. Sci.**USA* **114**, 201708558 (2017). 10.1073/pnas.1708558114 PMC5576829 28790187

[16] Kaushik AM (2021). Droplet-based single-cell measurements of 16S rRNA enable integrated bacteria identification and pheno-molecular antimicrobial susceptibility testing from clinical samples in 30 min. Adv. Sci..

[17] Rychert J (2019). Benefits and limitations of MALDI-TOF mass spectrometry for the identification of microorganisms. J. Infectiology.

[18] Ferreira L (2010). Direct identification of urinary tract pathogens from urine samples by matrix-assisted laser desorption ionization-time of flight mass spectrometry. J. Clin. Microbiol..

[19] de Roux A (2006). Mixed community-acquired pneumonia in hospitalised patients. Eur. Respir. J..

[20] Baltekin, Ö. et al. Evaluation of an ultra-rapid antibiotic susceptibility testing method on positive blood cultures with Escherichia coli. Preprint at *medRxiv*10.1101/2021.12.14.21267046 (2021).

[21] Kempf VAJ, Trebesius K, Autenrieth IB (2000). Fluorescent in situ hybridization allows rapid identification of microorganisms in blood cultures. J. Clin. Microbiol..

[22] O’Connor OM, Alnahhas RN, Lugagne J-B, Dunlop MJ (2022). DeLTA 2.0: A deep learning pipeline for quantifying single-cell spatial and temporal dynamics. PLoS Comput. Biol..

[23] Ronneberger, O., Fischer, P. & Brox, T. U-Net: Convolutional networks for biomedical image segmentation. *Lecture Notes Comput. Sci.*10.1007/978-3-319-24574-4_28 (2015).

[24] Stringer C, Wang T, Michaelos M, Pachitariu M (2020). Cellpose: A generalist algorithm for cellular segmentation. Nat. Methods.

[25] Cutler, K. J. et al. Omnipose: a high-precision morphology-independent solution for bacterial cell segmentation. *Nat Methods*10.1038/s41592-022-01639-4 (2022). 10.1038/s41592-022-01639-4 PMC9636021 36253643

[26] Waisman A, Norris AM, Elías Costa M, Kopinke D (2021). Automatic and unbiased segmentation and quantification of myofibers in skeletal muscle. Sci. Rep..

[27] Magnusson KEG, Jalden J, Gilbert PM, Blau HM (2015). Global linking of cell tracks using the Viterbi algorithm. IEEE Trans. Med. Imaging.

[28] Bertinetto, L., Valmadre, J., Henriques, J. F., Vedaldi, A. & Torr, P. H. S. Fully-convolutional siamese networks for object tracking. In *Computer Vision – ECCV 2016 Workshops* 850–865 (2016).

[29] Weng, X., Wang, Y., Man, Y. & Kitani, K. M. GNN3DMOT: Graph neural network for 3D multi-object tracking with 2D-3D multi-feature learning. In *2020 IEEE/CVF Conference on Computer Vision and Pattern Recognition (CVPR)* (2020).

[30] Wang J (2018). Bacterial species-identifiable magnetic nanosystems for early sepsis diagnosis and extracorporeal photodynamic blood disinfection. Nanoscale.

[31] Charnot-Katsikas, A. et al. Use of the accelerate pheno system for identification and antimicrobial susceptibility testing of pathogens in positive blood cultures and impact on time to results and workflow. *J. Clin. Microbiol.* **56**, e01166-17 (2017). 10.1128/JCM.01166-17 PMC5744213 29118168

[32] Opota O, Croxatto A, Prod’hom G, Greub G (2015). Blood culture-based diagnosis of bacteraemia: State of the art. Clin. Microbiol. Infect..

[33] Tabah A (2012). Characteristics and determinants of outcome of hospital-acquired bloodstream infections in intensive care units: The EUROBACT International Cohort Study. Intensive Care Med..

[34] EUCAST- European committee on antibiotic susceptibility testing. *Clinical breakpoints - breakpoints and guidance* https://www.eucast.org/clinical_breakpoints/ (2021).

[35] Lubeck E, Coskun AF, Zhiyentayev T, Ahmad M, Cai L (2014). Single-cell in situ RNA profiling by sequential hybridization. Nat. Methods.

[36] Chen, K. H., Boettiger, A. N., Moffitt, J. R., Wang, S. & Zhuang, X. RNA imaging. Spatially resolved, highly multiplexed RNA profiling in single cells. *Science* **348**, aaa6090 (2015). 10.1126/science.aaa6090 PMC4662681 25858977

[37] Zaborskyte, G., Andersen, J. B., Kragh, K. N. & Ciofu, O. Real-time monitoring of nfxB mutant occurrence and dynamics in pseudomonas aeruginosa biofilm exposed to subinhibitory concentrations of ciprofloxacin. *Antimicrobial Agents Chemother.* **61**, e02292-16 (2017). 10.1128/AAC.02292-16 PMC5328521 27993856

[38] Camsund D (2020). Time-resolved imaging-based CRISPRi screening. Nat. Methods.

[39] Edelstein AD (2014). Advanced methods of microscope control using μManager software. J. Biol. Methods.

[40] Alm EW, Oerther DB, Larsen N, Stahl DA, Raskin L (1996). The oligonucleotide probe database. Appl. Environ. Microbiol..

[41] Greuter D, Loy A, Horn M, Rattei T (2016). probeBase–an online resource for rRNA-targeted oligonucleotide probes and primers: New features 2016. Nucleic Acids Res..

