# Supplementary material for: Rapid antibiotic susceptibility testing and species identification for mixed samples
Source: Nat Commun. 2022 Oct 20;13:6215. doi: 10.1038/s41467-022-33659-1 (PMC9584937; doi:10.1038/s41467-022-33659-1)
Supplement: Supplementary file 1 — Supplementary Information [file 41467_2022_33659_MOESM1_ESM.pdf]

Supplementary Information

Rapid antibiotic susceptibility testing and species identification for mixed samples

Methods 1: Cell Segmentation: Omnipose and U-net training and performance

Dataset:

The cell segmentation training data contains 232 phase-contrast images and their corresponding labeled mask. Out of 232 images, 82 were randomly sampled phase-contrast images from mixed species experiments and 150 images contained only *E. coli* constitutively expressing fluorescent marker mVenus. Mixed-species phase-contrast images were hand labeled using ImageJ ROI manager, LabelsToROIs tool[1] and custom ImageJ script that converts regions to labeled images with each distinct region (cell) getting a unique label. Labels for the *E. coli* images were obtained by converting their corresponding fluorescent images into binary masks smoothing and adaptive thresholding. These images were curated to remove artifacts or miss-segmentations that occurred during the binary mask generation.

Omnipose training:

Omnipose network[2] predicts intermediate fields that are later used to reconstruct cell labels. **SI fig 1a** shows the 8 intermediate fields that are used for training the network, of which 4 (flow-x, flow-y, boundary and distance) are directly predicted by the network and the rest are used in loss function calculations. The network was trained on inputs of size 320x320 pixels using SGD optimizer and learning rate 0.05 for 1000 epochs. The augmentations were kept the same as the original paper[2]. 20% percent of data was used for validation of the model during training. The outputs of the network need to be post-processed to generate final labels. This reconstruction involves tracing the predicted flows to give per-pixel cell labels followed by spatial clustering step such as DBSCAN[3] on these noisy labels. We used a parallel version of DBSCAN[4] to improve the speed of cell segmentation as we found it to be a time-consuming step. **SI fig 1b** shows this improvement of 2x in speed achieved by parallelization of the clustering step. We measure Jaccard index vs IoU matching threshold as in Omnipose[2] and Cellpose papers[5]. **SI fig 1c** shows the performance of the network on a different subset of data i.e. mixed vs *E. coli*. We find that the network performance is similar across these 2 subsets.

U-net training:

A U-net that takes a phase-contrast image as input and predicts cell-vs-background maps[6] was shown to perform well on *E. coli* data in mother-machine channels. U-net requires a lot more labeled data than we have for mixed species images. To improve the loss values on our dataset we use a pre-training step on a larger dataset (2583 phase-contrast images and corresponding binary masks) containing only *E. coli.* Pre-training of U-net was done on images of size 320x320 pixels. The number of training examples used during one iteration of the training loop, i.e. batch size is 8. Each of these images were obtained by randomly cropping 320x320 pixels from the original images of large size (1024x800). These cropped images were scaled with a random ratio between 0.75 and 1.25, to cover various ranges of cell sizes and resized back to 320x320. Random noise, illumination adjustments, gaussian blurring, and rotation of the images were done on the training data pairs as described in[7]. Any operation that gave a non-binary value in the binary mask was thresholded using otsu thresholding. 20% percent of the training data was used for validation of the model. In addition to image augmentations, weight maps were generated using morphological operations to give more weight to the pixels separating cells in the loss function, as described in the original U-net paper[8]. **SI fig 1d** shows a sample input image, its corresponding binary mask and weight map used during the training process. The loss function used to train the net is dice-loss + weighted binary cross-entropy[9]. Adam optimizer [10] was used with a learning rate of 10^{-4} and the U-net was trained for 10 epochs until the losses flattened out. The weights were stored and used as initialization of new networks later.

After the pre-training step, we initialize a new U-net with pre-trained weights and train the network on the same dataset used for training the omnipose network. Augmentation and training of the network was similar to the pre-training. A learning rate of 10^{-5} was used and the network was trained until loss curves flatten out. As in the previous model we evaluate the network on both subset of data ie. mixed vs *E. coli.* **SI fig 1e** shows the comparison between different subsets. We can see that the U-net performance is as high as Omnipose for *E. coli* but a bit lower for the mixed species data.

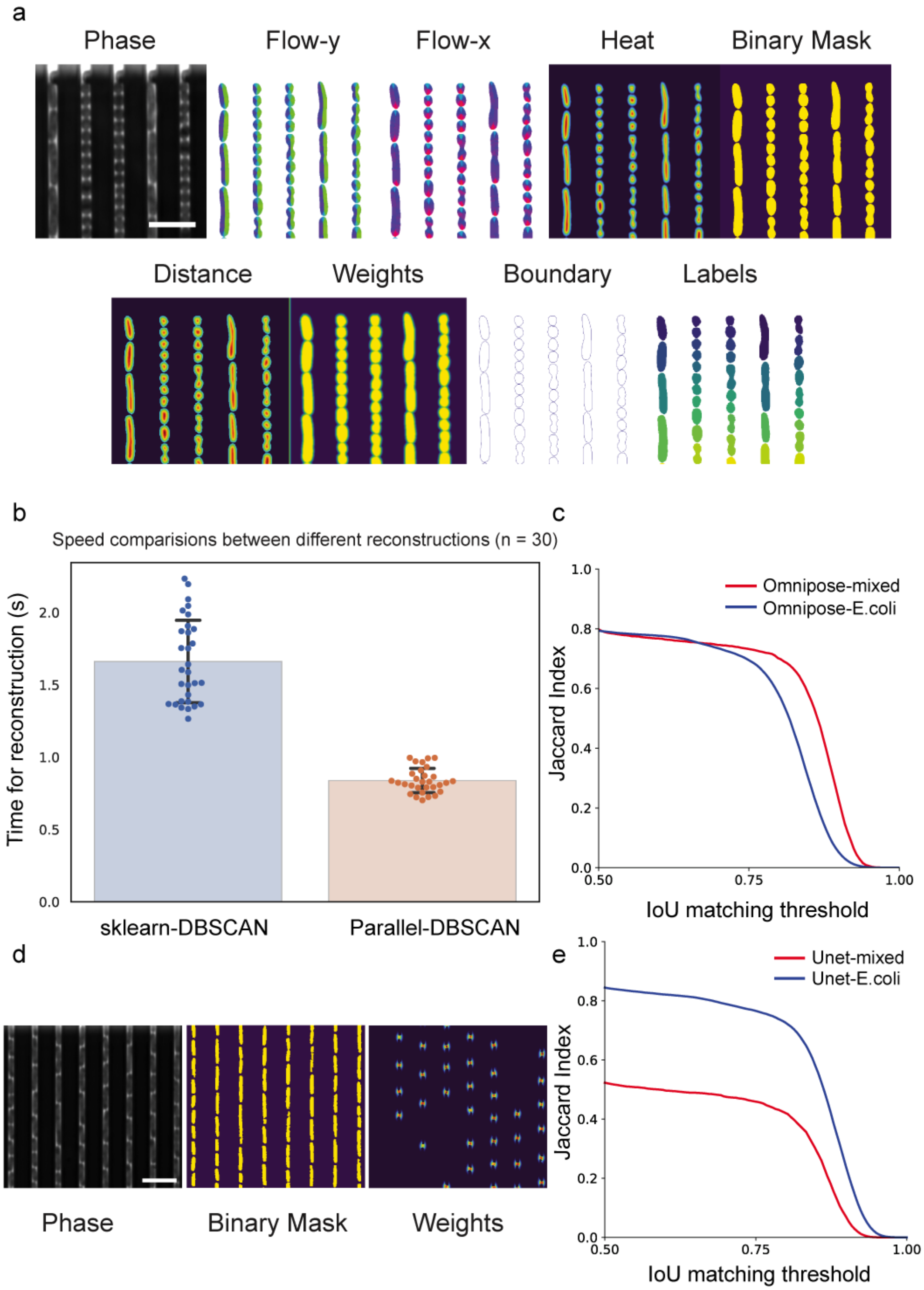

Supplementary Figure 1: *a) 8 fields used for training Omnipose network. Scale bar 5 µm. b) Plot comparing reconstruction speeds between two different methods on a set of images (n=30). Data shown is mean ± s.d. c) Performance comparison between E. coli and mixed datasets for Omnipose model d) Example of training data used for U-net. Scale bar 5 µm. e) Performance comparison between E. coli and mixed datasets for the U-net model.*

SI figure 2 shows the phase-contrast image and corresponding cell detection for each species loaded in separate chips. Omnipose network trained on our data was used on all images and the data has not been part of the training of the network.

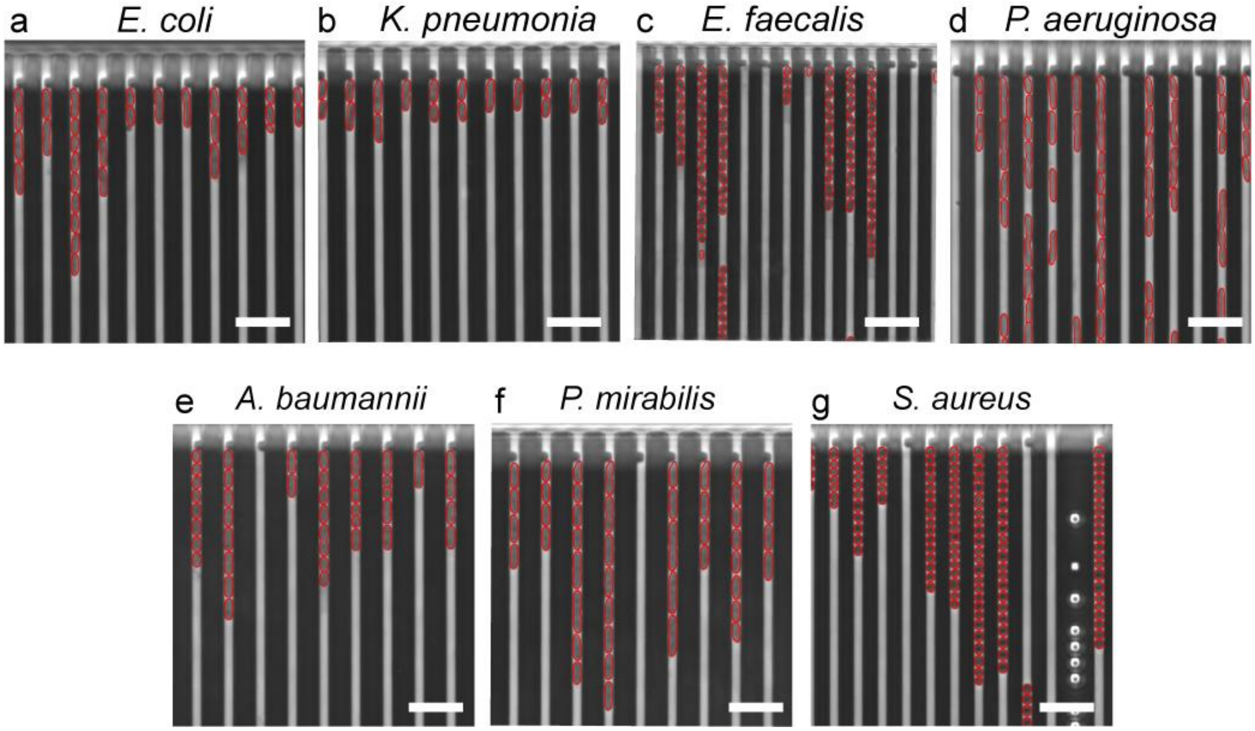

Supplementary Figure 2: *Segmentation mask from the cell segmentation network overlayed on phase-contrast images of individual species images. Scale bars in all images are 5 µm. a) E. coli b) K. pneumoniae c) E. faecalis d) P. aeruginosa e) A. baumannii f) P. mirabilis g) S. aureus. Each species segmentation was done on ~600 images from n=20 locations on the fluidic device. One image from each experiment is shown for illustration. Each experiment was performed once.*

Methods 2: Channel detection

Channel detection was done using the same U-net architecture as used for cell segmentation. The original U-net described in[8] has the number of feature channels varying from 64 at the input to 1024 before upsampling, doubling after every downsampling step. In this channel detection net, the number of the feature channels were reduced by a factor of 8 at all stages compared to the original U-net model. Training data was generated using image processing operations (histograms of intensity, peak finding on intensity histograms, binary morphological operations, etc). Dataset was manually curated to remove images whose channels couldn't be detected with histograms of intensity and peak finding operations. The same data augmentations and loss functions used for the cell segmentation model were used during training of the channel detection network (**SI section 1**). All pixels were given the same weight in the loss function. The training optimization is similar to that of the cell segmentation model training. **SI fig 3a** shows a sample of

the training data used as input for this net. **SI fig 3b**, shows the training and validation loss curves and **SI fig 3c** shows the raw phase-contrast image overlayed with contours of the channel segmentation.

Each phase-contrast image has a fixed number of channels in which cells grow. The mother machine channels were detected in each phase contrast image of the time-lapse microscopy data and location of each channel was mapped to its channel-number in each image. Using these locations time-series stacks of individual growth channel were constructed for further analysis. **SI fig 3d** shows the pixel offset distribution between channel locations detected using channel segmentation predictions and ground-truths of the test data not seen by the network.

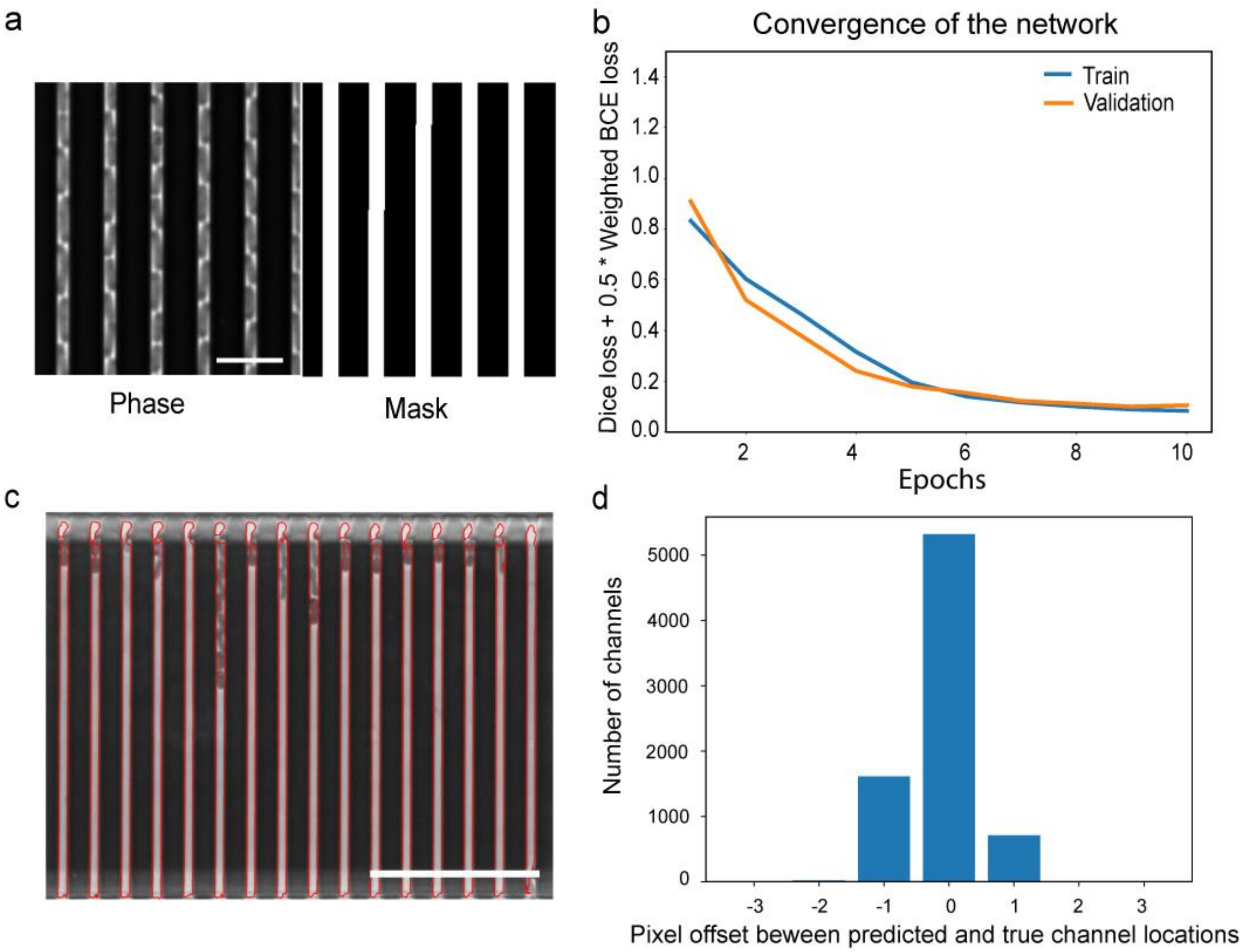

Supplementary Figure 3: *a) Sample training data for channel detection. Phase and binary mask example images. Scale bar 5 µm b) Training/validation loss curves c) Phase-contrast image and its corresponding channel segmentation mask. Scale bar, 20 µm. d) Pixel offset in channel locations between ground truths and predictions on test dataset (n=7760 channels total)*

Methods 3: Processing FISH Images with single color per species

Fluorescence images were acquired in 4 channels for the FISH-ID. Each FISH probe (**Supplementary data 1**) was designed to bind to a single species. Image processing operations like smoothing, histogram equalization was performed on the images and the background was subtracted using the empty channel values present in each image. Regions on each image containing signals above a threshold were detected and regions longer than 50 pixels were considered for species ID. The thresholds were set for each of the 4 fluorescence channel images individually during analysis to get the binary mask for each image. Thresholds were determined automatically using the Otsu method or set to 8000 (whichever is higher) after background subtraction. **SI fig 4** shows all the 4 fluorescent channel images with bounding boxes outlining the regions with signal.

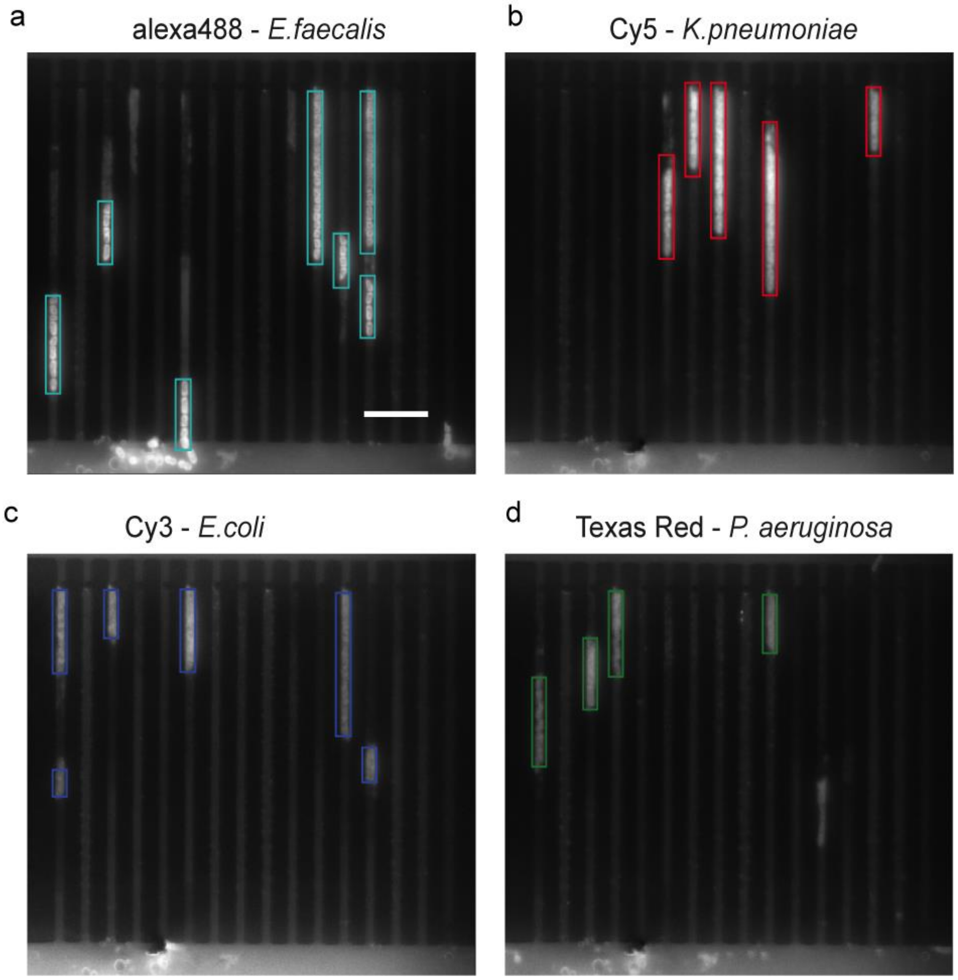

Supplementary Figure 4: *Four fluorescent channel images corresponding to each FISH probe/species with bounding boxes around regions where cells are. a) Alexa 488 - E. faecalis b) Cy5 - K.pneumoniae c) Cy3 - E.coli d) Texas Red - P.aeruginosa. Scale bar, 9µm. Bounding boxes were drawn for n>2000 growth channels (SI fig 9h) present in each experiment analyzed.*

Methods 4: Cell tracking

Cell tracking involves linking cells from one frame to the next while they are growing in the mother-machine channels. A cell from frame at time *t* was compared against all the cells in time frame *t*+1, predict the association between them into 3 categories: no-link, link of movement type, link of division type. The classification problem was formulated as an edge classification problem similar to the one described in[11]. The features of the cells (centroid, eccentricity, length, area, etc) were transformed using MLP (multi-layer perceptrons) and edge features were constructed using the features of the objects that are linked by it. Edge features were transformed again using an MLP. Loss function for training is a combination of Batch Triplet loss and affinity loss as in [11] but with a modification for predicting multiple types of associations. An overview of the network is shown in **SI fig 5a**.

To obtain training data for cell-tracking, we built a simulator that simulates the growth of cells inside mother-machine channels and keeps track of movements and divisions. The simulation involves constructing binary masks of cells whose growth rate is sampled from a distribution at each time step. At each time step, we update the image of the cell as well as its position in the growth channel. The type of link between two cells of a frame is stored during the simulation and used as ground truth. Cells that reach the end of the channel are dropped off from further timepoints. The parameters used for simulation such as cell-size, division size, growth rate and shape are chosen to emulate the real data. We use 1000 stacks of channels, with each stack containing 100 frames and their corresponding links as training data. **SI fig 5b** shows a sample stack of 30 frames generated by the simulator with two shapes of cells representing rod shaped and coccoid shaped cells.

When running the tracker network on experimental data, a cell from frame at time *t* was compared to all the cells at time *t+1* to obtain probabilities over the classes of edges. The class with the highest probability is used to describe the edge between cells that were compared. Links are set between two frames and tracks were constructed to generate cell lineages. Spurious links predicted by the networks (especially merge events due to segmentation errors) are cleaned during the track construction using IoU metric between the cells that are linked. Division events are also checked for IoU and area ratio between mother and daughter cells. Any link predictions that don't meet the IoU criteria are dropped from tracking. **SI fig 5c** shows the confusion matrices showing improvements in removing spurious links.

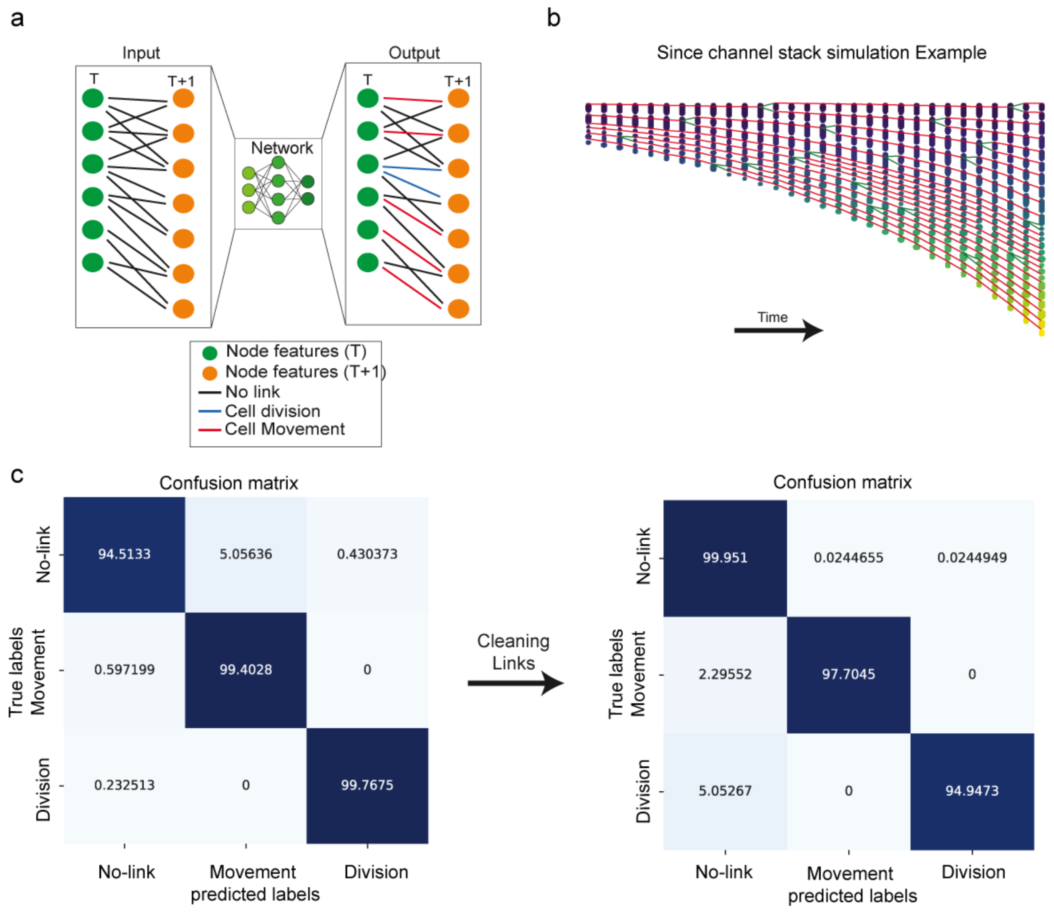

Supplementary Figure 5: *Cell tracking a) Overview of tracker network inputs and outputs. b) Example stack of training data produced by simulation. 1000 such stacks were used as training data. c) Confusion matrices showing improvements in removing spurious link predictions after IoU thresholding. True labels have 645006 No-links, 59163 movement links and 4040 division links.*

Methods 5: Species assignment for tracks using FISH data

The tracks ending in the last frame before fixing the cells were labeled with their species names obtained from the FISH data. This labeling was done based on which bounding boxes of the fluorescent data the centroids of the last blob in the track fell in. These species labels were rolled back in time to $t = 0$, thus labeling all the tracks since the beginning of the experiment.
SI fig 6 shows a few of these labeled tracks in different colors for different species.

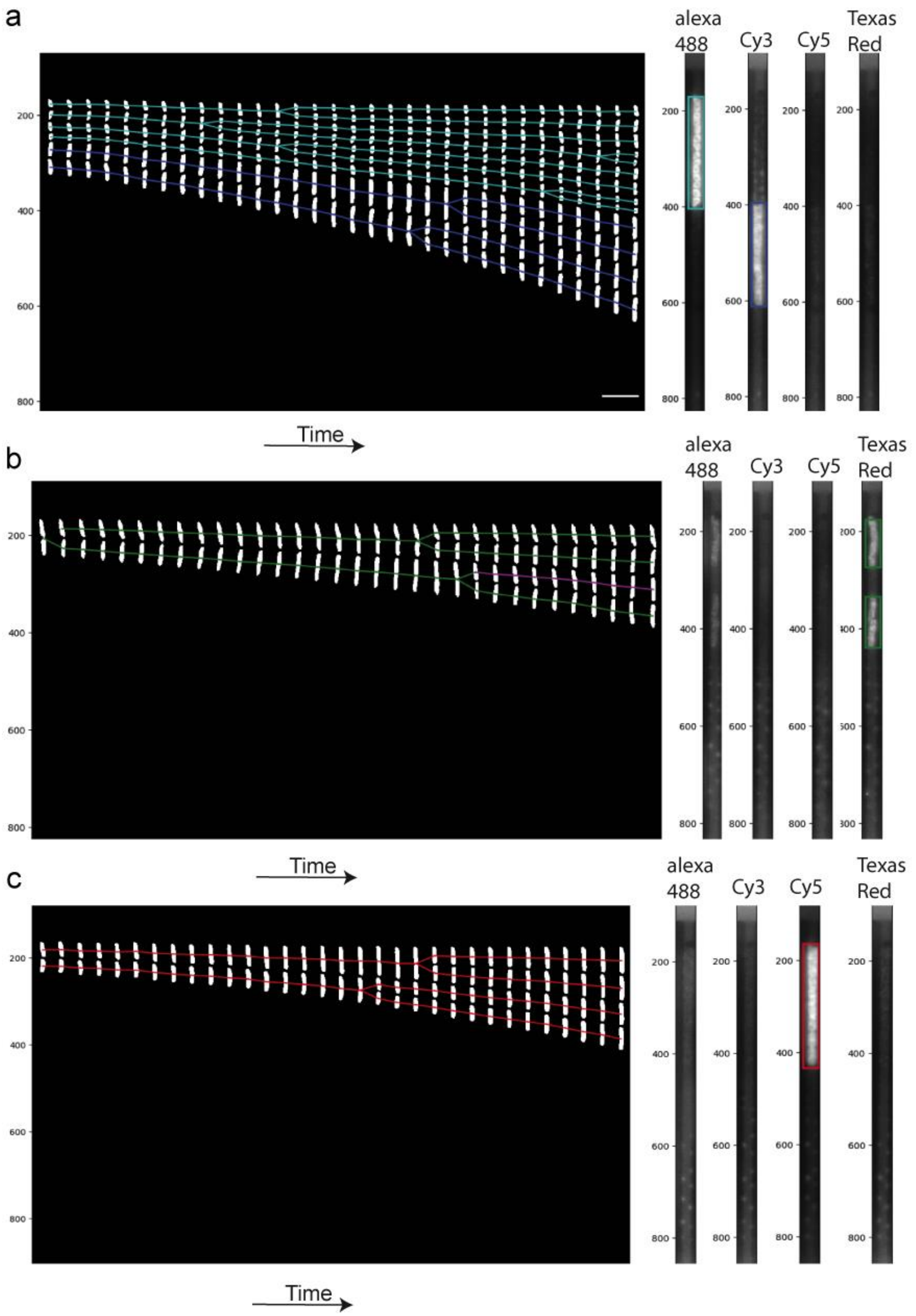

Supplementary Figure 6: *Tracks labeled with different colors for different species a) E. faecalis (cyan) and E. coli(blue) in the same channel b) P. aeruginosa (green) and track with undetermined species(magenta) c) K. pneumoniae(red). Scale bar, 5 µm for both panels. Time series stacks were constructed for n>2000 (SI Fig 9h) growth channels present in all experiments.*

Methods 6: Multi-Channel fluorescence Species assignment.

SI fig 7 shows images of single species loaded in different chips and the images in four fluorescent channels after performing combinatorial FISH assay. It can be seen from the figure that the combination of two probes (colors) represent one single species.

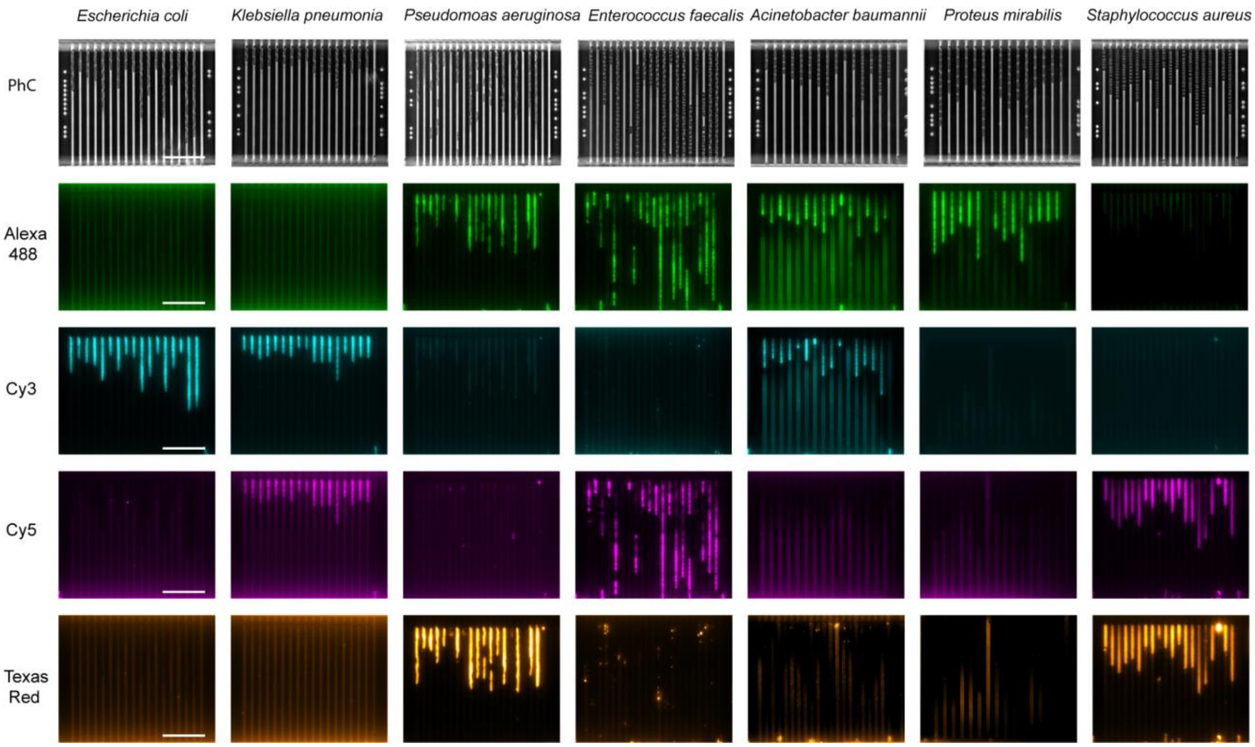

Supplementary Figure 7: *Example images of individual bacterial species when performed combinatorial FISH. After the hybridization step, images were captured in different channels (PhC, Alexa 488, Cy3, Cy5, and Texas Red channels). Depending on the detection probe hybridized to adapter sequences of the individual bacterial species, cells were expected to be visualized in two different channels. For example, Escherichia coli (Cy3), Klebsiella pneumonia (Cy3 and Cy5) Pseudomonas aeruginosa (Alexa 488 and Texas red), Enterococcus faecalis (Alexa 488 and Cy5), Acinetobacter baumannii (Alexa 488 and Cy3), Proteus mirabilis (Alexa 488), Staphylococcus aureus (Cy5 and Texas red). Scale bar, 20µm. Each experiment was performed once, but the same assay was done 8 times as shown in main text fig 5.*

In a typical experiment mixed species samples are loaded in the chip and combinatorial FISH assay is performed to get images such as the one included in the main text **(Fig 4b)**. As shown in **SI fig 7**, the adapter sequences and fluorescent probes are designed to bind to each species uniquely in two different fluorescent channels. To assign species labels to tracks a classification method that could classify the signal from the 4-dimensional fluorescence image stack is required. Training data for the classification method was created using a bounding box drawing tool LabelImg[12]. Data for each species was obtained by picking a few images from control experiments done with single species and experiments with 2-species loaded onto the microfluidic chip. All the data was pooled and balanced to obtain 30000 (4-dimensional) data points for each species. Data

for the background class was sampled randomly from the background of all the images in the dataset. We used the same concentration of fluorescent probes and imaging conditions in all the experiments. Signals from regions on the chip representing autofluorescence of PDMS were subtracted from the training data. Dataset was balanced to get the same number of data points in all the classes. We split the data into a test (30%) and train set (70%) and trained a random forest classifier using sklearn. **SI fig 8a** shows the classification accuracy and **SI fig 8b** shows the confusion matrix on the test data. We use the classifier to classify each pixel into 8-classes (7 for individual species with a known FISH signature, 1 for the background). **SI fig 8c** shows pixel-wise class assignment on fluorescence image stacks taken from each of the repeats of four experiments shown in main text Fig 4, i.e data from experiments not seen in training. We can see that the classification is in line with what is expected from each of these experiments i.e the classifier finds the species that were loaded in that particular experiment with very high accuracy. A small percentage of pixels can be misclassified, but the probability of finding a continuous cell-like region of these misclassified pixels is very low (**SI fig 8c**).

We use the growth channel location information of the last phase-contrast image before genotyping to extract fluorescence images slices corresponding to each growth channel. Bounding boxes over continuous regions on the image stack belonging to the same class of cells were calculated. Each bounding box should also be longer than 90 pixels, to count it as valid and use it for species assignment. The centroids of the tracks falling in these bounding boxes were labeled with the cell type of the predicted classes. **SI fig 8d** shows cell counts for all the cells detected in each of the 4 experiments on the reference side of the chip.

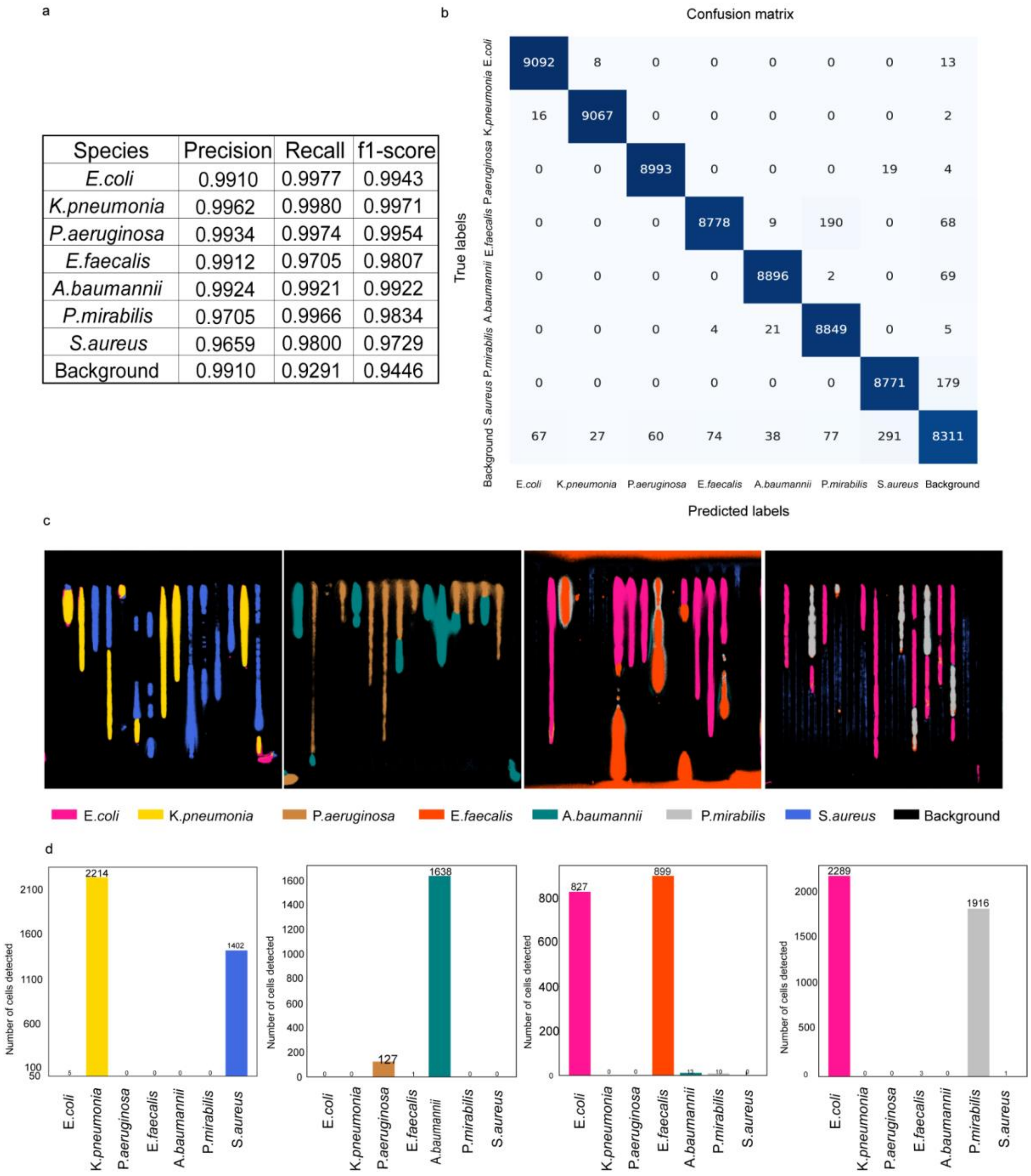

Species	Precision	Recall	f1-score
E.coli	0.9910	0.9977	0.9943
K.pneumonia	0.9962	0.9980	0.9971
P.aeruginosa	0.9934	0.9974	0.9954
E.faecalis	0.9912	0.9705	0.9807
A.baumannii	0.9924	0.9921	0.9922
P.mirabilis	0.9705	0.9966	0.9834
S.aureus	0.9659	0.9800	0.9729
Background	0.9910	0.9291	0.9446

Supplementary Figure 8: *Species assignment for multi-channel fluorescence a) Classification accuracy of 8 class classification. b) Confusion matrix of the classifier on test data c) Pixel-wise classification of multi-color experimental data from 4 experiments. d) Cell counts in the four experiments show the number of cells detected for each species.*

Methods 7: Growth Curve estimations

Growth rates were calculated by fitting exponential functions on the areas of cells in a single track on a sliding window of 5 frames, corresponding to 10 min. For each timepoint in the experiment, growth rates were collected from all the existing labeled tracks in that frame that existed for a 5 frame window. These growth rates were averaged to get the mean growth rate of that particular species. Growth rates were collected for both the reference and the antibiotic treated population. The normalized growth rate was calculated as the ratio of growth rates with respect to the treatment population. The plots in **SI fig 9a, 9b, 9c, 9d** show the normalized growth rates of all the four species used in an experiment along with their respective standard error of the mean. **SI fig 9e** shows the normalized growth rates of all 4 species plotted in the same graph for comparison. **SI fig 9f** shows the pooled normalized growth rates, i.e., growth rates without species identification. **SI fig 9g** shows the number of cells that went into tracking as a function of time and **SI fig 9h** shows the distribution of different species in the channels of the mother machine device.

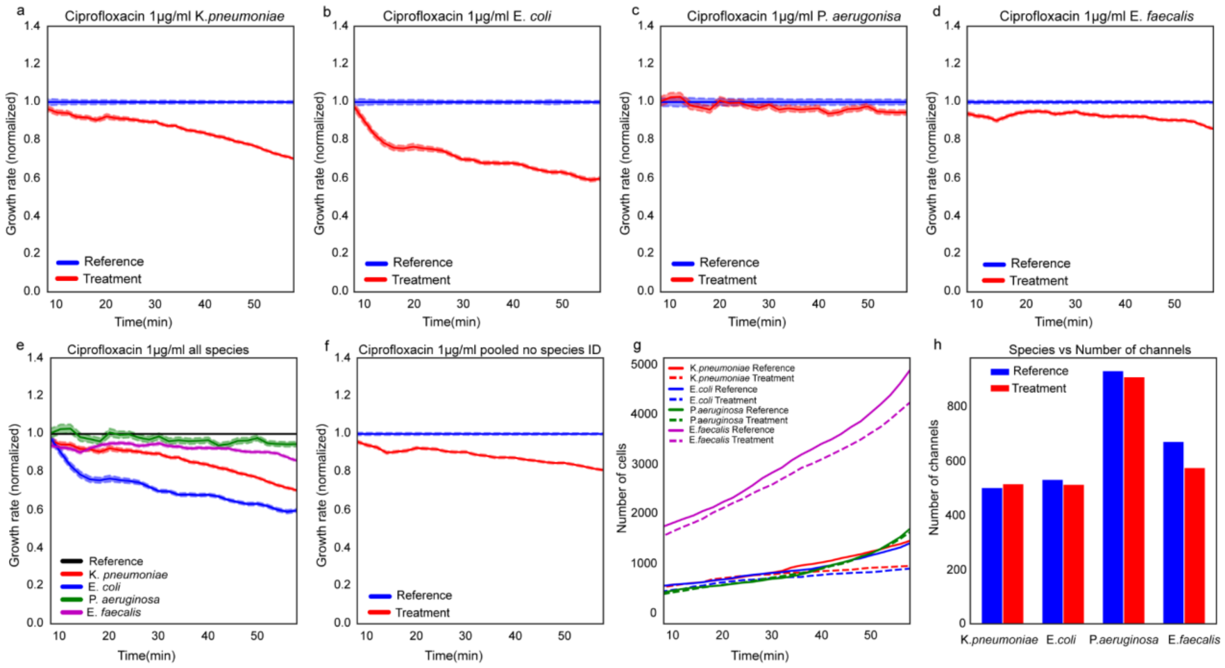

Supplementary Figure 9: *A panel showing results of one experiment a-d) Species-wise growth AST profiles for Ciprofloxacin 1μg/ml e) All species AST f) AST without species-ID g) Number of cells used to calculate growth rates as a function of time. f) Number of channels in which each species was detected.*

Methods 8: Loading differences between a chip and bulk cultures

We made comparisons of loading bias with various concentrations of *E. coli* and *E. faecalis* with respect to bulk cultures. Pure cultures were grown overnight from glycerol stocks and then diluted to 1:1000 times in a fresh medium, allowing them to grow until they reach the mid-exponential

phase. Cells were further diluted to an OD_{600} 0.05 and to calculate the number of cells in the bulk cultures at respective OD's, we performed a serial dilution and placed them on agar plates. We observed, in E*. coli*, on average 189 colonies corresponded to 1.9 x 10^6 CFU/ml and whereas in E*. faecalis,* 213 colonies corresponded to 2.1 x 10^6 CFU/ml. Next, we mixed them in various ratios of concentration and loaded them on the microfluidic chip. Results of these experiments were shown in supplementary data 5.

References

1. Waisman, A., Norris, A. M., Elías Costa, M. & Kopinke, D. Automatic and unbiased segmentation and quantification of myofibers in skeletal muscle. *Sci. Rep.* **11**, 1–14 (2021).
2. Cutler, K. J., Stringer, C., Wiggins, P. A. & Mougous, J. D. Omnipose: a high-precision morphology-independent solution for bacterial cell segmentation. *bioRxiv* 2021.11.03.467199 (2021) doi:10.1101/2021.11.03.467199.
3. DBSCAN. https://en.wikipedia.org/wiki/DBSCAN (2007).
4. Wang, Y., Gu, Y. & Shun, J. Theoretically-Efficient and Practical Parallel DBSCAN. *Proceedings of the 2020 ACM SIGMOD International Conference on Management of Data* (2020) doi:10.1145/3318464.3380582.
5. Stringer, C., Wang, T., Michaelos, M. & Pachitariu, M. Cellpose: a generalist algorithm for cellular segmentation. *Nat. Methods* **18**, 100–106 (2020).
6. O'Connor, O. M., Alnahhas, R. N., Lugagne, J.-B. & Dunlop, M. J. DeLTA 2.0: A deep learning pipeline for quantifying single-cell spatial and temporal dynamics. doi:10.1101/2021.08.10.455795.
7. Shorten, C. & Khoshgoftaar, T. M. A survey on Image Data Augmentation for Deep Learning. *Journal of Big Data* **6**, 1–48 (2019).
8. Ronneberger, O., Fischer, P. & Brox, T. U-Net: Convolutional Networks for Biomedical Image Segmentation. (2015).
9. Jadon, S. A survey of loss functions for semantic segmentation. *2020 IEEE Conference on Computational Intelligence in Bioinformatics and Computational Biology (CIBCB)* (2020)

doi:10.1109/cibcb48159.2020.9277638.

10. Kingma, D. P. & Ba, J. Adam: A Method for Stochastic Optimization. *arXiv [cs.LG]* (2014).

11. Weng, X., Wang, Y., Man, Y. & Kitani, K. M. GNN3DMOT: Graph Neural Network for 3D Multi-Object Tracking With 2D-3D Multi-Feature Learning. *2020 IEEE/CVF Conference on Computer Vision and Pattern Recognition (CVPR)* (2020) doi:10.1109/cvpr42600.2020.00653.

12. LabelImg. *GitHub* https://github.com/tzutalin/labelImg.
