# Supplementary material for: Rapid antibiotic susceptibility testing and species identification for mixed samples
Source: Nat Commun. 2022 Oct 20;13:6215. doi: 10.1038/s41467-022-33659-1 (PMC9584937; doi:10.1038/s41467-022-33659-1)
Supplement: Supplementary file 2 — Reporting Summary [file 41467_2022_33659_MOESM2_ESM.pdf]

nature portfolio

Corresponding author(s): Johan Elf

Last updated by author(s): Sep 16, 2022

Reporting Summary

Nature Portfolio wishes to improve the reproducibility of the work that we publish. This form provides structure for consistency and transparency in reporting. For further information on Nature Portfolio policies, see our Editorial Policies and the Editorial Policy Checklist.

Statistics

For all statistical analyses, confirm that the following items are present in the figure legend, table legend, main text, or Methods section.

n/a	Confirmed	
☐	☒	The exact sample size (n) for each experimental group/condition, given as a discrete number and unit of measurement
☐	☒	A statement on whether measurements were taken from distinct samples or whether the same sample was measured repeatedly
☒	☐	The statistical test(s) used AND whether they are one- or two-sided<br>*Only common tests should be described solely by name; describe more complex techniques in the Methods section.*
☒	☐	A description of all covariates tested
☒	☐	A description of any assumptions or corrections, such as tests of normality and adjustment for multiple comparisons
☐	☒	A full description of the statistical parameters including central tendency (e.g. means) or other basic estimates (e.g. regression coefficient) AND variation (e.g. standard deviation) or associated estimates of uncertainty (e.g. confidence intervals)
☒	☐	For null hypothesis testing, the test statistic (e.g. F, t, r) with confidence intervals, effect sizes, degrees of freedom and P value noted<br>*Give P values as exact values whenever suitable.*
☒	☐	For Bayesian analysis, information on the choice of priors and Markov chain Monte Carlo settings
☒	☐	For hierarchical and complex designs, identification of the appropriate level for tests and full reporting of outcomes
☒	☐	Estimates of effect sizes (e.g. Cohen's d, Pearson's r), indicating how they were calculated

Our web collection on statistics for biologists contains articles on many of the points above.

Software and code

Policy information about availability of computer code

Data collection	High-throughput microfluidic experiments were acquired Micro-manager 1.4 version. FISH images were labelled using labelImg 1.8.6. Raw images for cell segmentation were labelled using ImageJ (1.8.0_172 64bit) ROI manager and LabelsToROIs (v1.0).
Data analysis	We use custom image processing code with deep learning models and simple machine-learning based classifiers. The code is made publicly available on Github (https://github.com/karempudi/ASTFISH.git). All code is written in Python (>3.9.7) and all neural networks were built using PyTorch (v1.11.0). Analysis of each experiment was done in jupyter notebooks (v.6.4.5) provided with the code repository. Jupyter notebooks for replicating figures in the paper are also provided in the code repository. Parallel-DBSCAN (free software under MIT license) was used. Omnipose cell segmentation utilities (free software under non-commercial use license) was used.

For manuscripts utilizing custom algorithms or software that are central to the research but not yet described in published literature, software must be made available to editors and reviewers. We strongly encourage code deposition in a community repository (e.g. GitHub). See the Nature Portfolio guidelines for submitting code & software for further information.

Data

Policy information about availability of data

All manuscripts must include a data availability statement. This statement should provide the following information, where applicable:

- Accession codes, unique identifiers, or web links for publicly available datasets
- A description of any restrictions on data availability
- For clinical datasets or third party data, please ensure that the statement adheres to our policy

Raw microscopy data for all experiments shown in the paper are available at https://doi.org/10.17044/scilifelab.20969161. All analysis objects and code is also available at https://doi.org/10.17044/scilifelab.20969161.

Field-specific reporting

Please select the one below that is the best fit for your research. If you are not sure, read the appropriate sections before making your selection.

☒ Life sciences ☐ Behavioural & social sciences ☐ Ecological, evolutionary & environmental sciences

For a reference copy of the document with all sections, see nature.com/documents/nr-reporting-summary-flat.pdf

Life sciences study design

All studies must disclose on these points even when the disclosure is negative.

Sample size	Sample size such as number of cells used for growth rates is as many bacteria that can be imaged at the selected time resolution. Alteast 100 cells were used to calculate growth rates for each species.
Data exclusions	Data that produced cell segmentation errors were excluded from growth rate calculations based on rapid changes in areas. These data are typically a small fraction. Cell tracking outputs were also cleaned for errors. These exclusions are described in supplementary methods. For species assignment in combinatorial FISH experiments, cell with very low counts are ignored. These were typically small fractions as shown in supplementary methods.
Replication	For direct probe FISH experiments with 4 species the same assay was repeated at least one time for each antibiotic. Combinatorial probe FISH experiments were done using the same assay at least 10 times: All 7 species present in the microfluidic chip in only one experiment. All 2 species combination experiments were repeated twice. Control experiments for combinatorial probe with single species were done once.
Randomization	Not applicable to this work. In all the experiments, we only use laboratory strains of each species and only compare growth rates of individual species when exposed to media with and without different antibiotics.
Blinding	The experimental setup was the same for all the experiments. All analysis steps were automated to reduce potential human bias. Same cell segmentation and tracking models were used in all the experiments, both for treated and reference cells. Hence, blinding is not relevant.

Reporting for specific materials, systems and methods

We require information from authors about some types of materials, experimental systems and methods used in many studies. Here, indicate whether each material, system or method listed is relevant to your study. If you are not sure if a list item applies to your research, read the appropriate section before selecting a response.

Materials & experimental systems

n/a	Involved in the study	
☒	☐	Antibodies
☒	☐	Eukaryotic cell lines
☒	☐	Palaeontology and archaeology
☒	☐	Animals and other organisms
☒	☐	Human research participants
☒	☐	Clinical data
☒	☐	Dual use research of concern

Methods

n/a	Involved in the study	
☒	☐	ChIP-seq
☒	☐	Flow cytometry
☒	☐	MRI-based neuroimaging
