# Supplementary material for: Rapid antibiotic susceptibility testing and species identification for mixed samples
Source: Nat Commun. 2022 Oct 20;13:6215. doi: 10.1038/s41467-022-33659-1 (PMC9584937; doi:10.1038/s41467-022-33659-1)
Supplement: Supplementary file 3 — Description of Additional Supplementary Files [file 41467_2022_33659_MOESM3_ESM.pdf]

Title: Supplementary Data 1
Description: An excel file with all the oligonucleotide probe sequences used for FISH.

Title: Supplementary Data 2
Description: An excel file with all the detection probe sequences used for combinatorial FISH.

Title: Supplementary Data 3
Description: An excel file with all the oligonucleotide sequences used for the combinatorial FISH.

Title: Supplementary Data 4
Description: An excel file with all the barcode sequences used to link with the target sequence.

Title: Supplementary Data 5
Description: An excel file with loading ratios of E. coli and E. faecalis detected in microfluidic chip and bulk culture.

Title: Supplementary movie 1
Description: An example time-lapse movie of mixed species in microfluidics chip growing in MH media without any antibiotics (Top) and respective FISH image (bottom).

Title: Supplementary movie 2
Description: An example time-lapse movie of mixed species in microfluidics chip growing in MH media with an antibiotic Vancomycin (Top) and respective FISH image (bottom).

Title: Supplementary movie 3
Description: An example time-lapse movie of mixed species in microfluidics chip growing in MH media with an antibiotic Ciprofloxacin (Top) and respective FISH image (bottom).

Title: Supplementary movie 4
Description: An example time-lapse movie of mixed species in microfluidics chip growing in MH media with an antibiotic Gentamicin (Top) and respective FISH image (bottom).

Title: Supplementary movie 5
Description: An example time-lapse movie of mixed species in microfluidics chip growing in MH media with an antibiotic Nitrofurantoin (Top) and respective FISH image (bottom).
