# Supplementary material for: Rapid antibiotic susceptibility testing and species identification for mixed samples
Source: Nat Commun. 2022 Oct 20;13:6215. doi: 10.1038/s41467-022-33659-1 (PMC9584937; doi:10.1038/s41467-022-33659-1)
Supplement: Supplementary file 9 — Supplementary movie S1 [file 41467_2022_33659_MOESM9_ESM.pptx]

## Slide 1
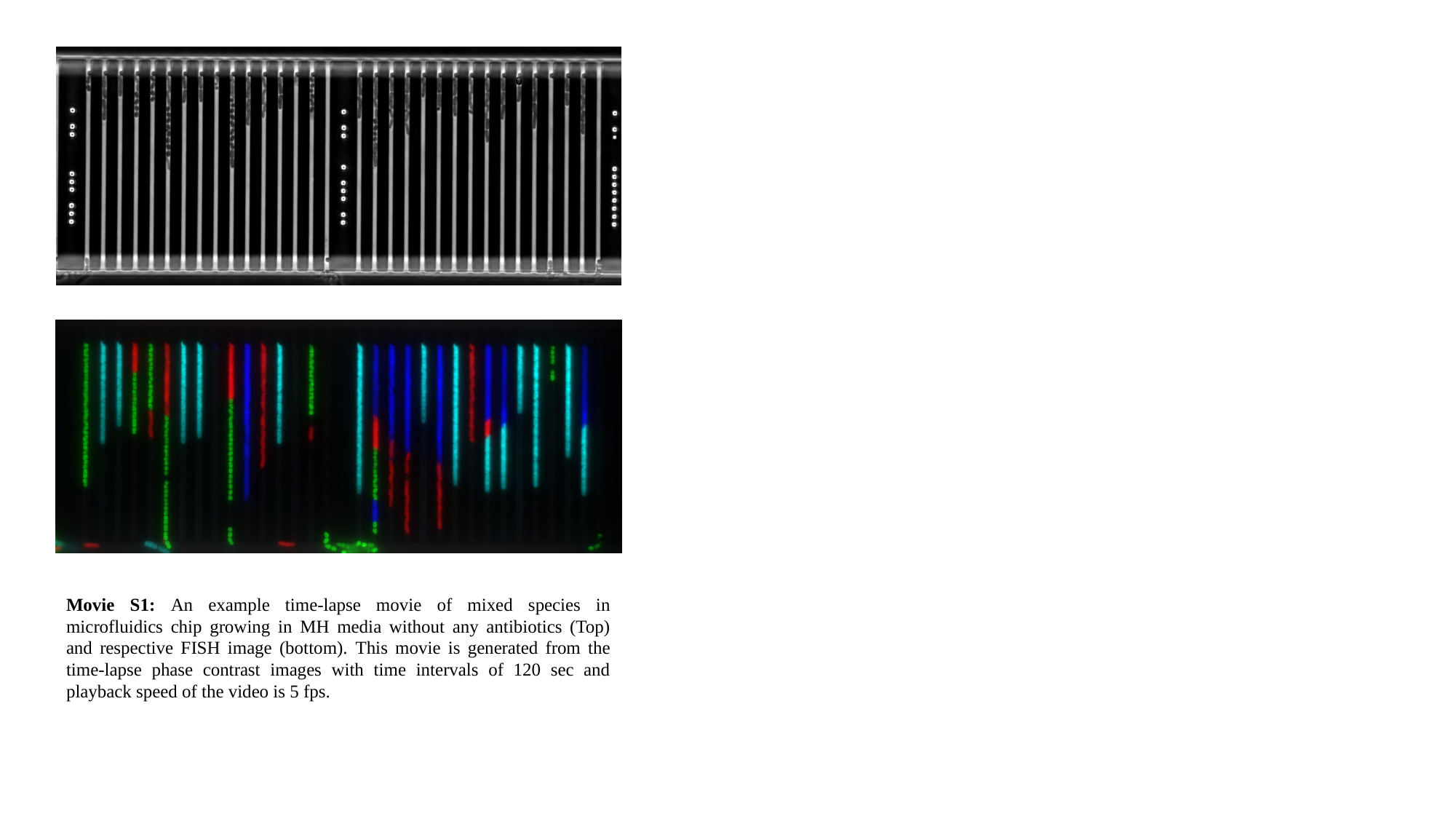

Movie S1: An example time-lapse movie of mixed species in microfluidics chip growing in MH media without any antibiotics (Top) and respective FISH image (bottom). This movie is generated from the time-lapse phase contrast images with time intervals of 120 sec and playback speed of the video is 5 fps.
